# Elevated plasma pentraxin-3 in polycystic ovary syndrome is associated with hyperandrogenism: a case-control study

**DOI:** 10.1186/s12902-021-00886-4

**Published:** 2021-12-03

**Authors:** Congcong Jin, Kexin Zou, Yue Xu, Haiyan Yang, Jiexue Pan

**Affiliations:** 1 The First Affiliated Hospital of Wenzhou Medical University, 325000 Wenzhou, China; 2 Obstetrics & Gynecology Hospital, Institute of Reproduction and Development, Fudan University, 200011 Shanghai, China; 3 Shanghai Key Laboratory of Embryo Original Diseases, 200030 Shanghai, China; 4 Shanghai Ji Ai Genetics and IVF Institute, Obstetrics and Gynecology Hospital, Fudan University, 200011 Shanghai, China

**Keywords:** Polycystic ovary syndrome, PTX3, Hyperandrogenism, Innate immunology

## Abstract

**Background:**

Pentraxin 3 (PTX3) - a crucial humoral innate immunity component – is related to obesity and cardiovascular complications in women who suffer from polycystic ovary syndrome (PCOS). However, the circulating PTX3 level in PCOS is still debated. In this study, we aimed to evaluate PTX3 plasma levels in PCOS women of childbearing age, and find possible endocrine/metabolic factors that could affect this level.

**Methods:**

A total of 360 women were enrolled: 120 PCOS women and 240 body mass index (BMI) matched normally ovulating women. Blood samples were collected on the third day of natural menstrual cycle or from the bleeding after progesterone withdrawal. The PTX3 concentration was measured by immunoassay.

**Results:**

The PTX3 plasma level was significantly higher in PCOS women compared to controls. There was a positive correlation between PTX3 plasma level and PCOS diagnosis, overweight, cycle length, serum LH to FSH ratio, estradiol, total testosterone (TT) on the third day of menstrual cycle, antral follicle count (AFC), as well as uric acid. Multivariant linear regression analysis indicated that participants’ serum PTX3 levels were proportional to the circulating TT level, existence of PCOS, basal estradiol level and AFC.

**Conclusions:**

Overall, the circulating PTX3 level was elevated in PCOS women and significantly associated with the presence of hyperandrogenism. This study provided the basis for further in-depth researches regarding PTX3 role in PCOS pathophysiology.

## Introduction

Polycystic ovary syndrome (PCOS) has multiple symptoms and is one of the most common endocrine disorders affecting women of child-bearing age [1]. Its prevalence worldwide varies from 5 to 19.9 % [2–4]. This syndrome is associated with hyperandrogenism, ovulatory dysfunction, decreased infertility, psychological discomfort [5] and increased incidence of metabolic abnormalities, such as insulin resistance and cardiovascular diseases [1]. Also, PCOS is a common cause of infertility, pregnancy complications, and unfavorable neonatal outcomes [6]. The heterogeneity of the condition leads to difficulties in etiology identification, and although several clinical trials and investigations have been conducted to comprehend its pathophysiological mechanisms [7], the details underpinning PCOS remain insufficiently understood.

Evidence supporting that PCOS is associated with chronic inflammatory state is emerging [8]. PCOS is associated with pro-inflammatory cytokines and chemokines elevation in plasma, such as interleukin-18 (IL-18) [9], monocyte chemoattractant protein-1 (MCP-1) [10, 11], and chemokine (C-C motif) ligand 3 (CCL3) [10]. Additionally, researchers have found an increased CD19^+^ B cells proportion and activity in PCOS women [12], which indicates a pathogenic role of lymphocytes in the development of the disease.

Innate immunity includes two facets: cellular and humoral. Pentraxin 3 (PTX3) is a fluid-phase pattern recognition molecule that belongs to the acute-reactants superfamily [e.g. C-reactive protein (CRP)]. It has many properties and can bind different molecules. Particularly, PTX3 is important for selected pathogens opsonization [13] and female fertility [14, 15]. Unlike the short pentraxin, PTX3 can be locally produced in response to inflammation by different cell types, including adipocytes [16], endothelial cells [17] and follicle cells [7]. Our previous study indicated that follicular cells from PCOS women have higher PTX3 expression [7]. Also, PCOS women with healthy weight presented higher ovarian PTX3 levels [18]. PTX3 is essential for the organization of cumulus oophorus extracellular matrix and fertilization [19, 20]. In mice, *Ptx3* gene deletion leads to female infertility and cumulus matrix instability [21]. PTX3 can also participate in PCOS occurrence [22–25] and its related metabolic complications [26]. However, PTX3 role in PCOS is still under dispute. We hypothesized that PTX3 level is altered in PCOS women. In the present study, we aimed to assess if plasma PTX3 level is associated with PCOS and investigate clinical and hormonal factors that would affect this level.

## Materials and methods

### Patient selection and sample collection

This case-control study was performed following the Declaration of Helsinki and approved by the Institutional Review Board of the First Affiliated Hospital of Wenzhou Medical University. Recently diagnosed PCOS (120) and normally ovulating women (240) were enrolled during January 2017 to August 2018 and gave written consent. All the subjects were referred to our reproductive department for infertility or pre-pregnancy checkup. PCOS patients were diagnosed according to the Rotterdam Consensus (European Society for Human Reproduction and Embryology/American Society for Reproductive Medicine criteria) [2]. All the PCOS cases in the present study had the ovulating dysfunction plus polycystic ovarian morphology after exclusion of specific identifiable disorders (adrenal hyperplasia, androgen-secreting tumors, hyperprolactinemia and Cushing’s syndrome). Additionally, each control woman met the following inclusion criteria: (1) normal ovarian volume; (2) menstrual cycle length between 26 and 33 days; (3) clinical, biochemical and sex hormonal profiles were within normal ranges; (4) no polycystic ovary morphology. All subjects in our study met the following inclusion criteria: (1) age between 21 and 35; (2) both ovaries present; (3) nonsmokers, normotensive, and not a regular consumer of alcoholic beverages; (4) did not have malign diseases, pelvic infections, chronic systemic diseases, and endometriosis diagnosed by vaginal ultrasound and/or laparoscopy; (5) none of the cases or controls was on any medications for at least 3 months prior the enrollment, including oral contraceptives, glucocorticoids, lipid-lowering, antiobesity, antidiabetes, antiandrogenic, and/-or ovulation-inducing agents.

**Fig. 1:**
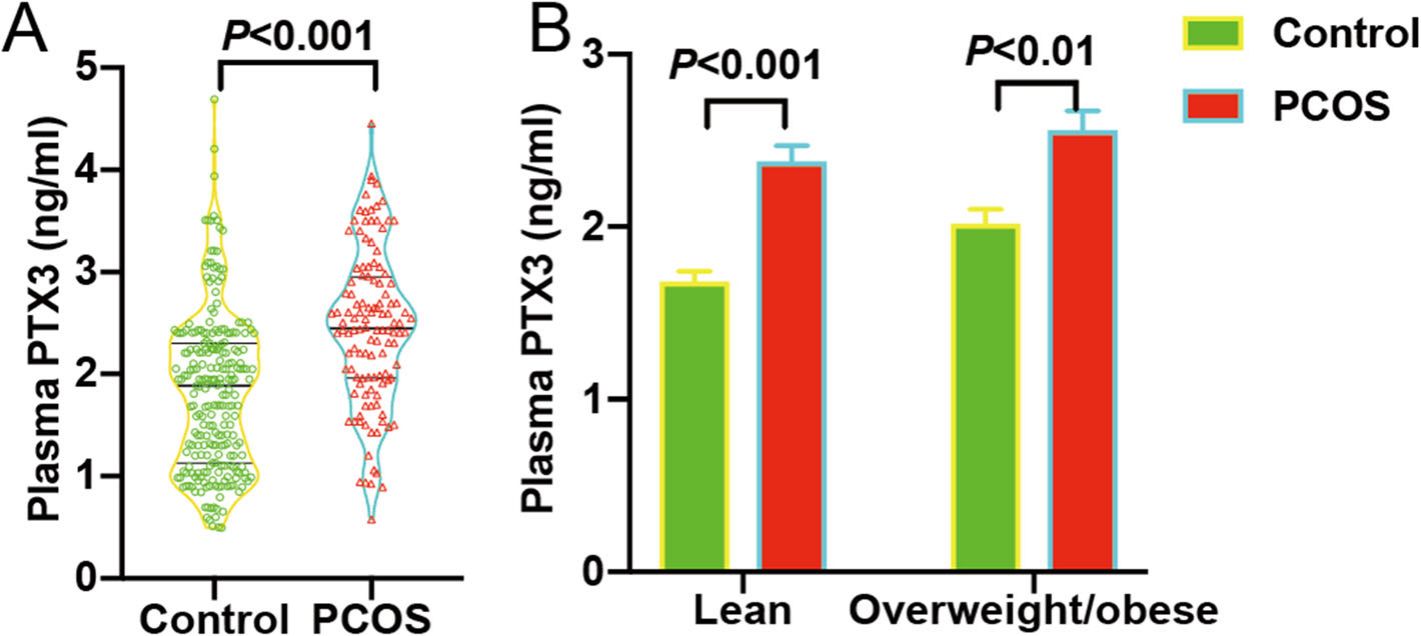
**A** Plasma PTX3 levels among normal ovulating women and PCOS subjects. The bottom and top of each box indicate the 25th and 75th percentiles, respectively. The line through the middle of each box represents the median. **B** Plasma PTX3 level in BMI-stratified women. *P* value was determined by an unpaired t-test

For each PCOS woman, two normally ovulating women with matched body mass index (BMI) were recruited as the control group (240) to minimize the effect of metabolic disturbances. The BMI was calculated as body-weight divided by the squared height (kg/m^2^). Finally, subjects were separated into different groups (Tables 1 and 2; Fig. 1B) according to their BMI. In general, Chinese females have a lower BMI and percentage of body fat than do white ones [27], so the BMI cut-off points for overweight/obesity are 23 kg/m^2^.

**Table 1:** Demographic data and clinical characteristics of enrolled subjects

Items		Control (*n*=240)		PCOS (*n*=120)		*P* value	
Age (years)		29.57±0.20		28.33±0.27		<0.001	
Body Mass Index (kg/m^2^)		22.38±0.22		22.54±0.30		0.658	
BMI < 18.5 (n [%])		28 [11.67 %]		14 [11.67 %]			
18.5 ≤ BMI < 23 (n [%])		112 [46.67 %]		56 [46.67 %]			
23 ≤ BMI < 30 (n [%])		96 [40 %]		48 [40 %]			
BMI ≥ 30 (n [%])		4 [1.67 %]		2 [1.67 %]			
Cycle length (days)		30.56±0.17		73.21±2.65		<0.001	
Day 3 LH/FSH		0.69±0.02		1.34±0.07		<0.001	
Day 3 TT (nmol/L)		1.28±0.03		1.71±0.05		<0.001	
Day 3 PRL (mIU/L)		315.75±8.11		295.20±10.29		0.131	
Day 3 E_2_ (pmol/L)		104.08±3.04		157.63±4.90		<0.001	
Antral follicle count		12.75±0.34		28.99±0.53		<0.001	
Fasting glucose (mmol/L)		5.26±0.03		5.26±0.05		0.906	
Uric acid (umol/L)		277.18±3.50		316.14±6.69		<0.001	
Creatinine (umol/L)		50.27±0.48		49.65±0.75		0.474	
Blood urea nitrogen (mmol/L)		4.09±0.07		4.18±0.09		0.416	
Bun/creatinine ratio		20.48±0.36		21.14±0.49		0.289	
ESR		14.88±0.70		16.34±0.99		0.228	
AST (U/L)		18.51±0.37		20.13±0.76		0.031	
ALT (U/L)		14.58±0.54		19.73±1.29		<0.001	
Total cholesterol (mmol/L)		4.50±0.05		4.64±0.08		0.106	
Triglyceride (mmol/L)		1.01±0.03		1.42±0.08		<0.001	
High-density lipoprotein (mmol/L)		1.46±0.18		1.37±0.32		0.004	
Low-density lipoprotein (mmol/L)		2.49±0.04		2.63±0.06		0.04	
Thyroid stimulating hormone (mIU/L)		2.32±0.08		2.35±0.11		0.843	
Free thyroxine (pmol/L)		10.91±0.08		11.35±0.14		0.032	
Free triiodothyronine (pmol/L)		4.63±0.04		4.77±0.06		0.004	
PTX3 (ng/ml)		1.82±0.05		2.46±0.07		<0.001	

Note: Day 3, the third day of spontaneous menstrual cycle; LH, luteinizinghormone; FSH, follicle stimulating hormone; TT, total testosterone; PRL, prolactin; E2, estradiol; ESR, erythrocyte sedimentation rate; ALT, alanine aminotransferase; AST, aspartate aminotransferase; PTX3, pentraxin 3

Values are presented as mean ± SE. Significance was determined by Student t tests

**Table 2:** Correlations of plasma PTX3 with each parameter

Items	*R*	*P* value
Existence of PCOS	0.363	<0.001
Age	-0.060	0.256
BMI > 23	0.171	0.001
Cycle length (days)	0.236	<0.001
Day 3 LH/FSH	0.269	<0.001
Day 3 TT	0.524	<0.001
Day 3 PRL	0.038	0.468
Day 3 E_2_	0.306	<0.001
Antral follicle count	0.244	<0.001
Fasting glucose (mmol/L)	-0.012	0.814
Uric acid (umol/L)	0.189	<0.001
Creatinine (umol/L)	0.054	0.309
Blood urea nitrogen (mmol/L)	-0.015	0.783
Bun/creatinine ratio	-0.049	0.356
ESR	0.035	0.506
AST (U/L)	0.095	0.072
ALT (U/L)	0.075	0.155
Total cholesterol (mmol/L)	-0.007	0.889
Triglyceride (mmol/L)	0.090	0.088
High-density lipoprotein (mmol/L)	-0.060	0.253
Low-density lipoprotein (mmol/L)	-0.009	0.865
Thyroid stimulating hormone (mIU/L)	-0.001	0.985
Free thyroxine (pmol/L)	0.014	0.792
Free triiodothyronine (pmol/L)	0.001	0.985

Data are presented as Pearson correlation coefficients

All subjects in our study met the following inclusion criteria: (1) age between 21 and 35; (2) both ovaries present; (3) nonsmokers, normotensive, and not a regular consumer of alcoholic beverages; (4) did not have malign diseases, pelvic infections, chronic systemic diseases, and endometriosis diagnosed by vaginal ultrasound and/or laparoscopy; (5) none of the cases or controls was on any medications for at least 3 months prior the enrollment, including oral contraceptives, glucocorticoids, lipid-lowering, antiobesity, antidiabetes, antiandrogenic, and/-or ovulation-inducing agents. Additionally, each control woman met the following inclusion criteria: (1) normal ovarian volume; (2) menstrual cycle length between 26 and 33 days; (3) clinical, biochemical and hormonal profiles were within normal ranges; (4) no polycystic ovary morphology.

All blood samples were collected on the third day of their natural menstrual cycle or from the bleeding after progesterone withdrawal. Five mL of venous blood were withdrawn in the morning between 8:00 and 9:00, after an overnight fast. Then, samples were centrifuged and the supernatants were stored at -80 °C until further analysis.

### Laboratory parameters determination

All blood samples were collected on the third day of their natural menstrual cycle or from the bleeding after progesterone withdrawal. Five mL of venous blood were withdrawn in the morning between 8:00 and 9:00, after an overnight fast. Then, samples were centrifuged and the supernatants were stored at -80 °C until further analysis.

Blood tests including hormones and liver functions were performed at the clinical laboratory of the First Affiliated Hospital of Wenzhou Medical University. The levels of Day 3 serum hormones were quantified using chemiluminescence assay (Unicel Dxl800, Beckman Coulter, USA). Fasting plasma glucose, serum triglycerides (TG), total cholesterol (TC), low-density lipoprotein (LDL), high-density lipoprotein (HDL) and hepatorenal function were measured by an autoanalyzer (AU5800, Beckman, USA). The plasma PTX3 levels were measured by The Quantikine Human Pentraxin 3/TSG-14 Immunoassay (R&D Systems, Inc. Minneapolis, MN, USA) at the same time, and using reagents from the same lot to reduce measurement variability.

### Statistical analysis

All statistical analyses were performed using the software SPSS version 24.0 (IBM Corp., Armonk, NY, USA). Continuous variables were noted as mean ± SE. For data comparison between groups with and without PCOS, an independent student’s t-test was used. The Pearson’s correlation coefficient was used to measure associations between plasma PTX3 and other variables. A multivariate analysis was performed using backward stepwise linearlogistic regression. All tests were two-sided and *P* values of 0.05 were considered statistically significant.

## Results

### Subjects’ baseline characteristics demographic data and clinical features

The characteristics of PCOS and control subjects are presented in Table 1. We compared the anthropometric and biochemical parameters between groups, and due to the matching criteria employed in this study, there was no significant BMI difference (Table 1). Among subjects, PCOS women were younger and characterized by significantly (*P* < 0.05) longer menstrual cycle, higher antral follicle count (AFC), serum luteinizing hormone (LH)/follicle-stimulating hormone (FSH) ratio, total testosterone (TT), and estradiol (E_2_) levels on the third day of menstrual cycle. Furthermore, compared with normally ovulating women, PCOS women had higher levels of uric acid, alanine aminotransferase (ALT), aspartate aminotransferase (AST), triglyceride, LDL, free thyroxine and free triiodothyronine, but lower HDL in the plasma. Among all subjects, 150 women were overweight/obese.

### Circulating PTX3 levels in PCOS women and controls

The circulating PTX3 level was significantly higher in PCOS (2.46 ± 0.07 ng/mL) than in control women (1.82 ± 0.05 ng/mL) (Table 1; Fig. 1 A), consisting in a marked elevation (30 %) (*P* < 0.001). BMI group analyses revealed that the mean PTX3 concentrations were higher in lean and overweight/obese PCOS women than the normally ovulating groups (Fig. 1B), especially the lean subjects whose BMI was < 23.

### Associations of enrolled parameters with circulating PTX3 levels

Pearson’s correlations were calculated to define parameters associated with plasma PTX3 levels (Table 2). Among all subjects, circulating PTX3 level had a positive relation with the existence of PCOS (*R* = 0.363, *P* < 0.001), overweight/obesity (*R* = 0.171, *P* = 0.001), menstrual cycle length (*R* = 0.236, *P* < 0.001), basal LH to FSH ratio (*R* = 0.269, *P* < 0.001), TT (*R* = 0.524, *P* < 0.001), and serum E_2_ (*R* = 0.306, *P* < 0.001), as well as AFC (*R* = 0.244, *P* < 0.001). Moreover, there was a positive correlation between plasma PTX3 level and uric acid (*R* = 0.189, *P* < 0.001). However, no associations were observed between the plasma PTX3 level and age, basal serum PRL and the remaining metabolic parameters.

The multiple linear logistic regression analyses were employed to explore the possible effects of variables on the circulatory PTX3 level (Table 3). After adjustment for basal LH/FSH, cycle length and uric acid, the plasma PTX3 level was significantly associated with basal TT (95 % CI = 0.493~0.785) and the existence of PCOS (95 % CI = 0.228~0.776). Additionally, basal E_2_ (95 % CI = 0.000~0.030) and AFC (95 % CI = -0.027~-0.001) showed weekly predictable values for plasma PTX3.

**Table 3:** Stepwise multiple regression analyses between plasma PTX3 and variables associated PTX3 in linear regression analysis

Variable	*Unstandardized coefficients*		*Standardized coefficients*	*P* value	*(95 %CI) for B*
	B	Std. Error	Beta		
Constant	0.959	0.146		<0.001	(0.671~1.247)
Day 3 TT	0.639	0.074	0.416	<0.001	(0.493~0.785)
Existence of PCOS	0.502	0.139	0.288	0.001	(0.228~0.776)
Overweight/obese or not	0.137	0.074	0.082	0.065	(-0.009~0.283)
Day 3 E_2_	0.002	0.001	0.108	0.030	(0.000~0.003)
AFC	-0.014	0.007	-0.159	0.036	(-0.027~-0.001)
Adjusted R^2^	0.322				
*P* value of the model	<0.001				
Std error of the estimate	0.677				

These factors were adjutsted in the multivariate regression analysis: BMI, Day 3 LH/FSH, cycle length and uric acid

## Discussion

Our study aimed to compare circulating PTX3 levels between BMI-matched PCOS and normally ovulating women. We observed a significant increase in plasma PTX3 levels of PCOS women. Meanwhile, TT level and the existence of PCOS were strongly associated with increased plasma PTX3.

PTX3 is an essential innate immunity component and increases in response to stress or inflammation. Based on its quaternary structure, PTX3 interacts with a series of ligands, playing multiple roles in different settings, such as cardiovascular diseases [28], fertility [14] and cancer [29]. In some inflammatory conditions, PTX3 has also been regarded as a biomarker for disease severity. Although both CRP and PTX3 are acute-phase proteins belonging to the pentraxin superfamily, the correlation between CRP and PTX3 levels was weak or absent in some conditions [30]. Unlike CRP, which is predominantly produced in the liver, PTX3 is synthesized in response to local inflammatory stimuli by a variety of cells including follicle cells [7, 18]. Several studies focusing on the chronic low-inflammatory state of PCOS indicated elevated CRP levels in this condition [31], and there were also several studies exploring the role of PTX3 in PCOS. However, the association between PTX3 and PCOS is still debated. The follicular PTX3 level among non-obese women was significantly higher in PCOS subjects and associated with the existence of PCOS and ovarian hyperandrogenism [18]. However, circulating PTX3 in PCOS women was previously reported as elevated [22, 23], diminished [24, 25], or similar [32] to non-PCOS subjects. Recently, researchers from Helsinki found that circulating PTX3 levels during *in vitro* fertilization could provide benefits in risk assessment for ovarian hyperstimulation syndrome [33]. Though the authors did not consider the existence of PCOS on the elevated PTX3 level, PCOS women are more predisposed to developing OHSS during the in vitro fertilization (IVF). This may provide explanation as the higher circulating PTX3 levels in our study. Katarzyna et al. have identified PTX3 as an endothelial dysfunction marker in young PCOS women [26]. Tosi et al. found that plasma PTX3 was decreased in PCOS women [24], but the enrolled PCOS subjects had significantly higher BMI (median BMI = 28.5) than non-PCOS ones (median BMI = 21.0). The differential BMI could be the possible explanations for the disaccord with our study. The correlations between PTX3 and BMI have been discussed in several studies, but there is no conclusive result [22, 25]. Therefore, it is of great significance to evaluate the plasma PTX3 level between BMI-matched PCOS and non-PCOS women. In our current study, the PCOS group and the controls were BMI-matched to minimize the effect of fat mass percentage and related metabolic disturbances on plasma PTX3 level. The average PTX3 level was 1.82 ± 0.05 ng/mL in normally ovulating women, whereas in PCOS women it was 2.46 ± 0.07 ng/mL. The association analysis (Table 2) revealed a positive association between obese/overweight status and plasma PTX3. However, the association attenuated after multivariant regression analysis was introduced. This could be due to the fact that the majority of subjects in our study were lean.

Although we have matched the BMI levels, there were significant differences in several metabolic parameters between the two groups (Table 1). However, we did not find any correlation between plasma PTX3 and these parameters, except for uric acid. Multiple regression analyses revealed that only circulating androgen level and the existence of PCOS were significantly correlated with could be the main predicable variables for plasma PTX3.

Most PCOS women suffer from hyperandrogenism [7, 34], and the PCOS women in this study also showed higher serum TT levels. There is evidence of a suppressive role of androgen on immune reactions [35], and the higher autoimmune diseases prevalence in women also indicates its immunosuppressive effect [36]. While Wang et al. suggested that exposure to hyperandrogenism might stimulate chronic ovarian inflammation [37], and Ashcroft et al. reported an immuno-enhancing effect of testosterone on macrophages [38]. Additionally, *in vivo* and *in vitro* studies also suggested that androgen excess stimulates inflammatory response in PCOS women [39]. Despite androgen being an immunoregulator, the potential role of hyperandrogenism on women’s the immune response, especially innate immunity has never been fully illustrated. PTX3 is synthesized by different cells including adipocytes [16], endothelial cells [17] and follicle cells [7], and is involved in PCOS metabolic complications. Previous study has found that PCOS women had higher PTX3 expression in follicle cells [7], and aberrant DNA modification and hyperandrogenism might be possible pathogenies. The results in this study also indicate that hyperandrogenism might induce PTX3 overproduction. However, whether elevated circulting PTX3 is an indicator of the severity of innate immunity or is a compensatory mechanism of hyperandrogenism, and the mechanisms related androgen-induced higher PTX3 expression requires further study.

The present study also has limitations. First, although we have matched the two groups with BMI, most women from China are relatively thin and most subjects enrolled in our study were non-obese, even the PCOS ones. And most PCOS women suffered from insulin resistance. It is of significance to confirm our results in more subjects, obese and non-obese; and insulin resistance should also be measured. Second, we measured the PTX3 level only during follicular phase and further studies should consider PTX3 variations during different menstrual stages. Finally, all the enrolled subjects were relatively young.

## Conclusions

We found that PCOS women had markedly increased PTX3 levels. Also, hyperandrogenism and the existence of PCOS were valuable predictors for elevated PTX3. This suggested that androgen may be a mediator in the pathogenesis of chronic low-grade inflammation for PCOS women.

## Data Availability

The datasets used or analysed during the current study are available from the corresponding author on reasonable request.
